# Development of a one-step reverse transcription-quantitative polymerase chain reaction assay for the detection of porcine reproductive and respiratory syndrome virus

**DOI:** 10.1371/journal.pone.0293042

**Published:** 2023-10-16

**Authors:** Hansong Chae, Hyun Soo Roh, Young Mi Jo, Won Gyeong Kim, Jeong Byoung Chae, Seung-Uk Shin, Jung Won Kang

**Affiliations:** R&D Center of Animal Technology, Animal Industry Data Korea, Gangnam-gu, Seoul, South Korea; Cairo University Faculty of Veterinary Medicine, EGYPT

## Abstract

Porcine reproductive and respiratory syndrome (PRRS) caused by PRRS virus (PRRSV) is an important disease that severely affects the swine industry and, therefore, warrants rapid and accurate diagnosis for its control. Despite the progress in developing diagnostic tools, including polymerase chain reaction (PCR)-based methods such as reverse transcription quantitative PCR (RT-qPCR) to diagnose PRRSV infection, its diagnosis at the genetic level is challenging because of its high genetic variability. Nevertheless, RT-qPCR is the easiest and fastest method for diagnosing PRRSV. Therefore, this study aimed to develop an RT-qPCR assay for rapid and accurate diagnosis of PRRSV by encompassing all publicly available PRRSV sequences. The developed assay using highly specific primers and probes could detect up to 10 copies of PRRSV-1 and -2 subtypes. Furthermore, a comparison of the performance of the developed assay with those of two commercial kits widely used in South Korea demonstrated the higher efficiency of the developed assay in detecting PRRSV infections in field samples. For PRRSV-1 detection, the developed assay showed a diagnostic agreement of 97.7% with the results of ORF5 sequencing, while for commercial kits, it showed 95.3% and 72.1% agreement. For PRRSV-2, the developed assay showed a diagnostic agreement of 97.7%, whereas the commercial kits showed 93% and 90.7% agreement. In conclusion, we developed an assay with higher accuracy than those of the tested commercial kits, which will contribute markedly to global PRRSV control.

## Introduction

Porcine reproductive and respiratory syndrome (PRRS) caused by PRRS virus (PRRSV) was first recognized in the 1980s and is characterized by reproductive failure, respiratory diseases, and elevated mortality rates in pigs [1–7]. The PRRSV is an RNA virus with a single-stranded genome. With an exceptionally high mutation rate (4.71 × 10^2^–9.8 ×10^2^/synonymous sites/year), the PRRS virus exhibits strain diversity in geographical regions and countries [8–10]. Despite this extensive genetic diversity, PRRSV can be classified into two major genotypes: PRRSV-1, previously known as Type 1 or European type, represented by the Lelystad virus strain, and PRRSV-2, previously known as Type 2 or North American type, represented by the VR-2332 strain. PRRSV-1 and -2 share approximately 70% nucleotide identity at the genomic level; however, PRRSV-1, more prevalent in Europe and Africa, has a higher genetic diversity and shows more frequent genetic recombination than PRRSV-2. On the contrary, PRRSV-2, with a lower diversity and a more stable genome, is predominant in North America and East Asia [11–17].

Because of the highly contagious nature of PRRSV and its resistance to moderate temperature, the disease has spread across numerous pig-producing countries, affecting virtually all regions except Australia, New Zealand, Scandinavia, and Switzerland [6, 12, 18–23]. This wide prevalence has not only raised concerns about the health and well-being of pig populations but has also had far-reaching implications on international trade involving pork products and has become a significant global concern in the swine industry [1–7]. The economic impact of PRRS is estimated at approximately 100 billion won in South Korea and approximately 600 million dollars in the United States [5, 6]. Therefore, research interests in detecting and diagnosing PRRSV for disease control and eradication have increased, leading to the evolution and reporting of many identification methods.

Earlier, the samples for the diagnosis of PRRSV infection were commonly obtained from whole blood; however, in consideration of animal welfare, a range of non-invasive sampling techniques, including collection of oral fluid, nasal swabs, and fecal samples, have been reported more recently to identify PRRSV [24–28]. Experimentally, these methods can be categorized into virus culture, antibody-based methods, such as enzyme-linked immunosorbent assay (ELISA), indirect immunofluorescence assays, immunoperoxidase monolayer assay, and methods targeting specific PRRSV genes. Moreover, genetic testing methods based on polymerase chain reaction (PCR), such as reverse transcriptase PCR (RT-PCR), quantitative RT-PCR (RT-qPCR), digital PCR, and next-generation sequencing have gained interest [19, 29–31]. Among these diagnostic methods, detecting viral RNA in whole blood using RT-qPCR is the most powerful and simple. This molecular diagnostic tool provides remarkable sensitivity and specificity, enabling the detection of low levels of viral RNA in samples. Therefore, RT-qPCR is highly useful for early detection and monitoring of PRRSV outbreaks. Additionally, the ability to quantify the amount of viral RNA in a sample allows for a more accurate assessment of infection severity [32–34].

In this study, we aimed to develop a reliable and efficient diagnostic tool to detect PRRSV-1 and -2 using RT-qPCR. The RT-qPCR assay was developed based on conserved regions of the PRRSV genome, allowing the detection of both PRRSV types with high sensitivity and specificity. Additionally, we assessed and compared the performance efficacy of the developed assay with those of two commercially available assays widely used in South Korea. This study will contribute to developing a reliable and efficient diagnostic tool for PRRSV detection, which is critical for controlling and eradicating this economically devastating virus.

## Materials and methods

### Primer and probe design

Among the PRRSV genome sequences registered in the National Center for Biotechnology Information (NCBI, http://www.ncbi.nlm.nih.gov) database, 1,024 genomes with accurate Open Reading Frame (ORF) annotation information were selected as analysis targets (sequences with partially registered ORF information were not selected). The selected genomes (n = 1,024) comprised 110 PRRSV-1 and 914 PRRSV-2 genomes. All selected genome sequences of PRRSV were aligned using MUSCLE (version 3.8.31) [35] and compared. The regions exhibiting a high degree of conservation (> 95% sequence homology among the total aligned sequences) within the PRRSV-1 (n = 110) and -2 (n = 914) genomes were identified.

Subsequently, PRRSV-1 and -2 specific primers and probes were designed from these identified conserved regions to diagnose PRRSV-1 and -2 strains, respectively. The possibility of self-and heterodimer formation was calculated using the Oligo Analyzer Tool (Integrated DNA Technologies, Coralville, IA, USA), and all primer and probe sets were tested for potential cross-reactivity with unrelated organism sequences using BLAST tools (https://blast.ncbi.nlm.nih.gov/Blast.cgi) at NCBI.

### Viruses and RNA extraction

A total of 211 whole blood samples were obtained from 13 pig farms in South Korea between March 2022 and March 2023, and the samples were stored at −80°C with RiboEx^TM^ LS (GeneAll Biotechnology, Daejeon, South Korea) until further analysis.

The reference PRRSV and other viruses used as commercial vaccines (and their sources) used for ORF Sanger sequencing, phylogenetic analysis, and assessment of specificity and sensitivity of the developed RT-qPCR assay are shown in Tables 1 and 2. These reference viruses and vaccines included Unistrain^®^ (HIPRA, Girona, Spain) and Porcilis^®^ (Merck Sharp &Dohme, NJ, USA) for PRRSV-1; Ingelvac^®^ PRRS MLV (Boehringer Ingelheim, Ingelheim, Germany) and Fostera^®^ (Zoetis Inc., NJ, USA) for PRRSV-2; SuiShot^®^ PTR2 (Jungang Vaccine, Daejeon, South Korea) for other pig viruses; and Inforce 3^®^ (Zoetis Inc., NJ, USA) and CattleMaster^®^ 4 (Zoetis Inc., NJ, USA) for bovine virus.

**Table 1:** Field samples and vaccines.

Field samples^a^ or vaccine		No. of samples tested	Origin
1	Unistrain^®^	1	HIPRA
2	Porcilis^®^	1	Merck Sharp &Dohme
3	Ingelvac^®^	1	Boehringer Ingelheim
4	Fostera^®^	1	Zoetis Inc.
5	PRRSV type 1 field samples	35/211^b^	13 different farms in South Korea
6	PRRSV type 2 field samples	81/211^b^	
7	PRRSV type 1/2 field samples	7/211^b^	
8	PRRSV negative field samples	88/211^c^	

^a^Tested using commercial RT-qPCR Kit A.

^b^The number of types of PRRSV samples/total number of field samples.

^c^The number of negative field samples tested/total number of field samples.

**Table 2:** Other viruses and vaccines for evaluation of the specificity.

Host		Organism or vaccine	Origin
1	Porcine	Porcine circovirus 2	KVCC-VR1900011
2	Porcine	Swine influenza virus	KVCC-VR2200031
3	Porcine	*Mycoplasma hyopneumoniae*	Field samples ^a^
4	Porcine	Porcine epidemic diarrhea virus	KVCC-VR1900041
5	Porcine	Transmissible gastroenteritis virus	KVCC-VR1600041
6	Porcine	Porcine rotavirus	KVCC-VR1700002
7	Porcine	Enterotoxigenic *Escherichia coli*	Field samples ^a^
8	Porcine	Shiga toxin-producing *Escherichia coli*	Field samples ^a^
9	Porcine	Salmonella typhimurium	KVCC-BA1900016
10	Porcine	SuiShot^®^ PTR2	Jungang Vaccine
11	Bovine	Inforce 3^®^ vaccine	Zoetis
12	Bovine	CattleMaster^®^ 4+VL5	Zoetis
13	Chicken	PoulShot^®^ QX-IB	Jungang Vaccine
14	Chicken	PoulShot^®^ Laryngo	Jungang Vaccine

^a^All field samples were collected from farms in South Korea and identified using targeted-specific PCR methods for each organism.

Hybrid-R blood RNA (GeneAll Biotechnology, Daejeon, South Korea) was used for total RNA extraction. For each extraction, a 250 μL whole blood sample was used and eluted in 50 μL. All field samples and reference virus RNA were stored at −80°C until further experiments.

### ORF5 sequencing and phylogenetic analysis

To confirm the presence of PRRSV, 43 of the 211 field samples were analyzed using ORF5 gene sequencing. Sequencing primers were designed for ORF4 and ORF6 to cover the entire ORF5 region (Table 3). PCR amplification was performed using an OPUS CFX Thermal cycler (Bio-Rad, Hercules, CA, USA). To generate cDNA, reverse transcription (RT) was performed using the PrimeScript RT reagent kit (Takara, Shiga, Japan) according to the manufacturer’s protocol. For the PCR step, each assay mixture contained 10 μL 2x TOPsimple DyeMIX-nTaq (Enzynomics, Daejeon, South Korea), 1 μL (0.5 uM final concentration) of each primer, and 1 μL cDNA (100 ng/ μL) template, and 7 μL distilled water (DW) in a final volume of 20 μL. The PCR mixture was processed with the following conditions: pre-denaturation at 95°C for 5 min, followed by 40 cycles of 95°C for 30 s, 60°C for 30 s, 72°C for 30 s, and final extension at 72°C for 5 min. Gel electrophoresis was performed using a 2% agarose gel at 100 V for 30 min to visualize the PCR amplicons (S1 Fig). The PCR products were confirmed using Sanger sequencing (Bioneer, Daejeon, South Korea).

**Table 3:** Primers used for PRRSV ORF5 Sanger sequencing.

Organism	Target region	Primer	Sequence	Amplicon size
PRRSV-1	ORF4-6	PRRSV1_F	ATGAGGTGGGCYACAACCAT	655 bp
		PRRSV1_R	TCTADGYYTSCCATTGYTC	
PRRSV-2	ORF4-6	PRRSV2_F	GGGCRACYGTTTTAGCCTGTC	649 bp
		PRRSV2_R	ACCCCATHGYTCYGCWGRA	

^a^ All primers were newly designed for this study

The referenced ORF5 sequences of PRRSV-1 strains, including those of Lelystad (M96262.2), Porcilis (MT311646.1), Unistrain (GU067771.1), and Ingelvac (KT988004.1); PRRSV-2 strains, including those of VR2332 (EF536003.1), Ingelvac (AF066183.4), PRRSV ATP (DQ988080), Prime pac (DQ779791.1), and Prevacent (KU131568.1); and the sequencing data of 43 field samples were used for the phylogenetic analysis. The maximum likelihood distance method of the MEGA X software (version 10.2.6) [36] was used with a bootstrap of 1,000 repetitions to construct the phylogenetic tree. Based on the ORF5 sequence, a strain was categorized as vaccine-like if the PRRSV reference or vaccine sequence shared more than 97.5% similarity with the PRRSV sequence obtained from the field sample. Conversely, if the similarity was below 97.5%, it was classified as a field strain [37].

### Establishment of RT-qPCR assay condition

For the RT-qPCR, each assay mixture comprised the following components: 6.24 μL GoTaq® Probe qPCR and RT-qPCR Systems master mix (Promega, Madison, WI, USA), PRRSV-1 and -2 specific primers and probes, each at a final concentration of 0.42 μM, and *GAPDH* primers and probe, each at a final concentration of 0.17 μM, 5 μL RNA template, and the final volume was adjusted to 12 μL using DW. The RT-qPCR amplification was performed using an OPUS CFX Thermal Cycler (Bio-Rad Laboratories). The thermal cycling conditions were as follows: an RT step at 50°C for 15 min, followed by 45 cycles of 95°C for 15 s, and 60°C for 30 s. Fluorescence signals were collected during the annealing step after each cycle. The RT-qPCR assay set the threshold for all fluorescence signals at 100, and a sample with a cycle threshold (Ct) ≤ 40 cycles was defined as positive.

### Analytical sensitivity and specificity of the RT-qPCR assay

To estimate the limit of detection (LoD) of the developed RT-qPCR, three types of standard plasmids were synthesized by Bionics in South Korea. Two plasmids included standard constructs for PRRSV-1 and -2, which were used for the complete ORF6-7 region sequence of Lelystad (M96262) and VR2332 (U87392), respectively. Furthermore, another plasmid was generated for *sus scrofa* GAPDH (AF017079), intended for use as an internal control. The standard plasmids were quantified using Nanodrop (Thermo Fisher, Waltham, MA, USA), and their respective copy numbers were calculated using the DNA Copy Number and Dilution Calculator (Thermo Fisher, Waltham, MA, USA). The plasmids were serially diluted ten-fold from 10^8^ to 1 plasmid copy (approximately 0.4 ng to 0.04 fg per reaction) and used to determine the LoD and RT-qPCR efficiency. The assessment of RT-qPCR efficiency was calculated by analyzing the amplification results through the Bio-Rad CFX Maestro 1.1 software (version 4.1). This analysis encompassed the evaluation of standard curves and amplification graphs, which collectively enabled the calculation of RT-qPCR efficiency.

Specificity was tested using nine representative porcine pathogens and one porcine viral diarrhea vaccine (porcine epidemic diarrhea virus, PEDV; transmissible gastroenteritis virus, TGEV; and porcine pseudorabies virus, PRV), two chicken vaccines (infectious bronchitis and laryngotracheitis), and two calf respiratory vaccines (bovine respiratory syncytial virus, BRSV; infectious bovine rhinotracheitis, IBR; bovine parainfluenza-3, PI3; and bovine viral diarrhea virus, BVDV) (Table 2).

### Performance evaluation of the developed RT-qPCR assay

To assess the reliability and efficiency of the RT-qPCR assay developed in this study, we performed a comparative analysis with two commercially available RT-qPCR assays (designated as Kit A and Kit B; Table 4) approved by the Korean Animal and Plant Quarantine Agency (KAPQA) and used ORF5 sequencing for verification.

**Table 4:** Details of the commercial RT-qPCR kits.

Commercial kit^a^	Method	Targets	Country	Interpretation of results
Kit A	Multiplex RT-qPCR	PRRSV 1, PRRSV 2	South Korea	Texas red Ct < 35: PRRSV-1 positive HEX Ct < 35: PRRSV-2 positive
Kit B	Multiplex RT-qPCR	PRRSV-1, PRRSV-2	South Korea	FAM Ct <40: PRRSV-1 positive HEX Ct <40: PRRSV-2 positive

^a^These commercial RT-qPCR assays are approved by the KAPQA in South Korea as a veterinary medical device.

A total of 43 samples were analyzed using all three methods, and the performance of the developed RT-qPCR assay was evaluated by comparing the results with those of commercially available kits using the remaining 68 samples.

Next, the diagnostic agreement between various test methods was quantified using the Percent Agreement, a commonly used metric for test comparison [38–40]. For instance, with PRRS-1 as a consideration, if tests X and Y are applied as diagnostic methods, the percent agreement (%) of X with Y can be calculated as follows:

Percentageagreement(%)=100*(A+D)/N=100*(a+c+I+k+f+n+h+p)/N (1)

See Tables 5 and 6 for the descriptions of the parameters in Eq 1.

**Table 5:** Description of the parameters in the metric (a–p) used to compare the two diagnostic tests X and Y (in Eqs 1–3).

Test Y (Reference)	Test X				Total
	Positive 1*	Positive 2^σ^	Positive 3^#^	Negative	
Positive 1 ^a^	a	b	c	d	
Positive 2 ^b^	e	f	g	h	
Positive 3^c^	i	j	k	l	
Negative	m	n	o	p	
Total					N

*PRRSV-1 positive.

^σ^PRRSV-2 positive.

^**#**^PRRSV-1 and -2 positive.

**Table 6:** Description of the parameters in the metric (A–D) used to compare the two diagnostic tests X and Y (in Eqs 1–3).

Test Y (Reference)	Test X		Total
	Positive	Negative	
Positive	A (a + c + i + k or f + g + j + k) ^a^	B (b + d + j + l or e + h + i + l) ^a^	A + B
Negative	C (e + g + m + o or b + c + n + o) ^a^	D (f + n + h + p or a + m + d + p) ^a^	C + D
Total	A + C	B + D	N

^a^Refer to Table 5.

The sensitivity and specificity of the tests were calculated following a previously described method [40]. Briefly, to determine the sensitivity and specificity for PRRSV-1, PRRSV-2 positive and negative samples were classified as negative, while samples with a co-infection of PRRSV-1 and 2 were considered PRRSV-1 positive. The sensitivity of test X in relation to test Y was obtained as follows:

Sensitivity=A/(A+B)*100=(a+c+i+k)/(a+c+i+k+b+d+j+l)*100 (2)

Similarly, the specificity of test X compared with that of test Y is calculated using the following equation:

Specificity=D/(C+D)*100=(f+n+h+p)/(e+g+m+o+f+n+h+p)*100 (3)

For a more comprehensive kit comparison, we additionally calculated Positive (PPV) and Negative Predictive Values (NPV) following previous studies [38–40] using the following equations:

PPV=A/(A+C)*100=(a+c+i+k)/(a+c+i+k+e+g+m+o)*100 (4)

NPV=D/(B+D)*100=(f+n+h+p)/(b+d+j+l+f+n+h+p)*100 (5)

## Results

### Design and interpretation of the developed RT-qPCR assay

The RT-qPCR utilized a three-target multiplex assay (Table 7 and S2 Fig). The assay was validated in *in-silico* using 1,024 PRRSV strains to determine whether the primers and probes accurately detected the target sequences. Given the high mutation rate observed in PRRSV, identifying sequence regions exhibiting perfect conservation and specificity posed a considerable challenge. Our approach was to identify the most conserved sequence regions while excluding regions with more than one base mismatch within sequences ranging from 18 to 25 bases. The criterion for a base match was that all aligned bases were at least 95% identical. Using the filtered sequences, the designed primers and probes showed high specificity, with no mismatches observed between the target sequences and the primers or probes. Moreover, none of the designed primers or probes showed significant cross-reactivity or homology with unrelated sequences. The threshold for the three fluorescence values was 100; if the result was less than 40 cycles, it was considered positive (Table 8).

**Table 7:** Primers and probes used in the developed RT-qPCR assay.

Organism	Gene	Name	Sequence ^a^
PRRSV-1	ORF6–7	1_F	CCTGCCCAYCACGTAGAAAG
		1_P	FAM- TACGCTGTGAGAAAGCCCGG -BHQ1
		1_R	AGTGCCGTTYACTGANGTY
PRRSV-2	ORF6–7	2_F	TGCCCACCACGTYGARAG
		2_P	HEX-TAACCACGCATTYGTCGTYCGGCG -BHQ1
		2_R	GCACCARTGTGCCGTTRAC
*Sus scrofa*	GAPDH	G_F	ACCTCCACTACATGGTCTACA
	[41]	G_P	Cy5- AGTATGATTCCACCCACGGCAAGT -BHQ2
		G_R	ATGACAAGCTTCCCGTTCTC

**Table 8:** Results criteria of the developed RT-qPCR kit.

	PRRSV-1 (FAM)	PRRSV-2 (HEX)	Internal control (Cy5)	Diagnostics result
1	FAM Ct < 40	HEX Ct < 40	Cy5 Ct < 40	PRRSV-1 and -2
2	FAM Ct < 40	HEX Ct > 40	Cy5 Ct < 40	PRRSV-1
3	FAM Ct > 40	HEX Ct < 40	Cy5 Ct < 40	PRRSV-2
4	FAM Ct > 40	HEX Ct > 40	Cy5 Ct < 40	Negative
5	FAM Ct < or > 40	HEX Ct < or > 40	Cy5 Ct > 40	Re-test^a^

^a^If the Ct value of Cy5 is > 40, the RNA was re-extracted from the sample, or the developed RT-qPCR was re-run.

### Analytical sensitivity and specificity of the developed RT-qPCR assay

**Fig 1:**
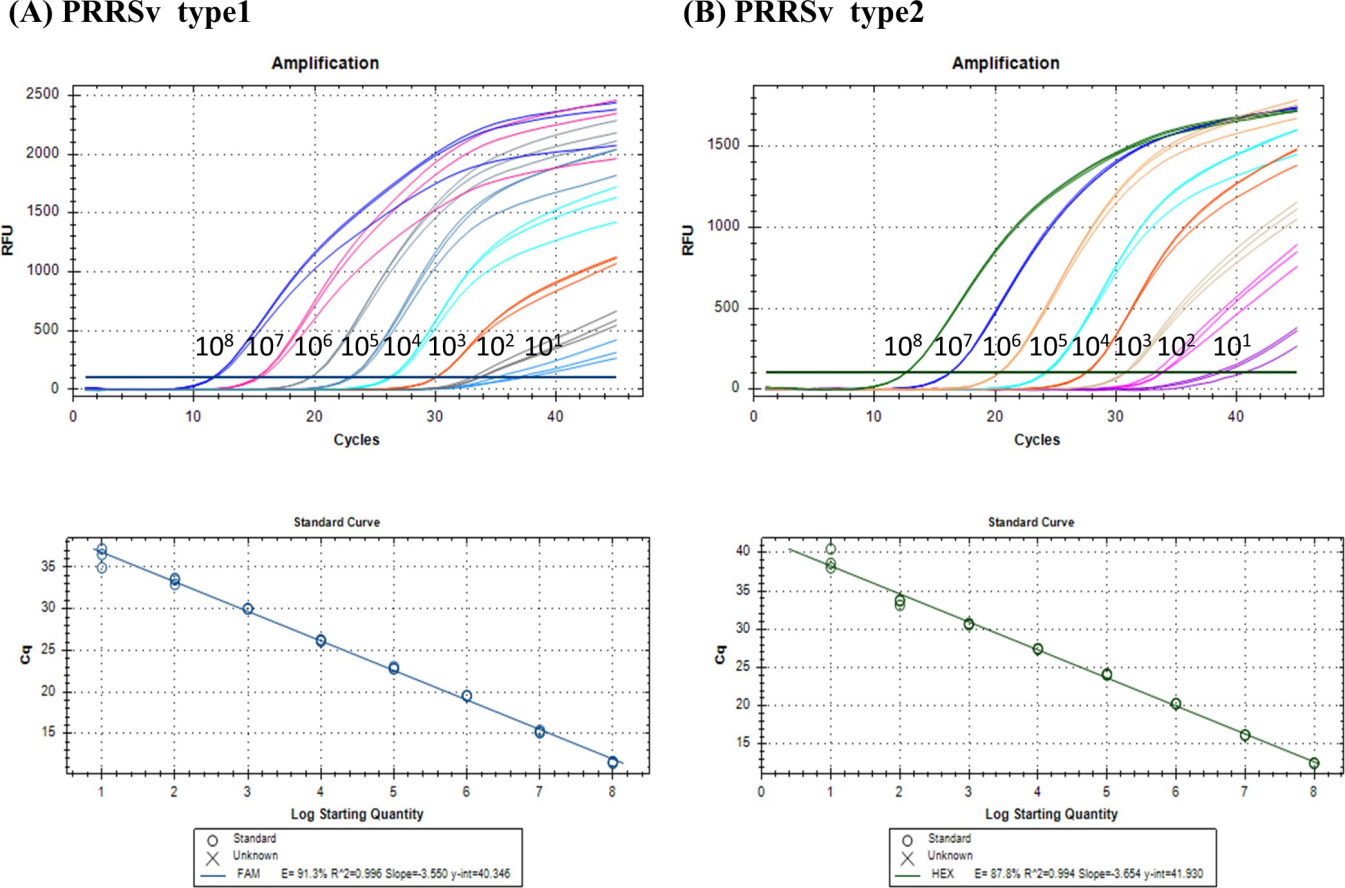
The analytic sensitivity results and standard curve of the developed RT-qPCR. The developed RT-qPCR’s amplification graphs and standard curves were generated using ten-fold dilutions of plasmid (1–10^8^ copies). (A) PRRSV-1 and (B) PRRSV-2.

The sensitivity of RT-qPCR was assessed based on the number of nucleotide copies. The LoD was determined to be ten copies of the plasmid, and the assay demonstrated a linear detection range of 1–10^8^ plasmid copies/reaction for PRRSV-1 and -2 (Table 9 and Fig 1). All RT-qPCR assays were performed in triplicate, and the mean values were used to generate standard curves. The efficiency of the RT-qPCR assay was 0.996 for PRRSV-1 and 0.994 for PRRSV-2 (Table 10 and Fig 1). These results indicate that the developed RT-qPCR assay is highly sensitive and capable of detecting low levels of viral nucleic acids, making it a valuable tool for PRRSV diagnosis. In addition, the specificity test confirmed that the results were negative for organisms other than PRRSV (Table 2), with a specificity of 100% for both PRRSV-1 and -2.

**Table 9:** Results of the analytic sensitivity.

Plasmid copies/reaction	Average Ct values	
	PRRSV-1	PRRSV-2
10^8^	10.39	11.90
10^7^	14.81	15.89
10^6^	19.51	20.25
10^5^	22.49	23.44
10^4^	25.59	26.94
10^3^	29.94	30.62
10^2^	33.86	34.03
10^1^	36.82	39.96
10^0^	N/A	N/A

**Table 10:** Results of the developed RT-qPCR’s standard curves.

Target	Efficiency	Linearity	Slope	LoD
PRRSV-1	91.3	0.996	-3.55	10 copies
PRRSV-2	87.8	0.994	-3.654	10 copies

### Evaluation of the developed RT-qPCR using field samples

**Fig 2:**
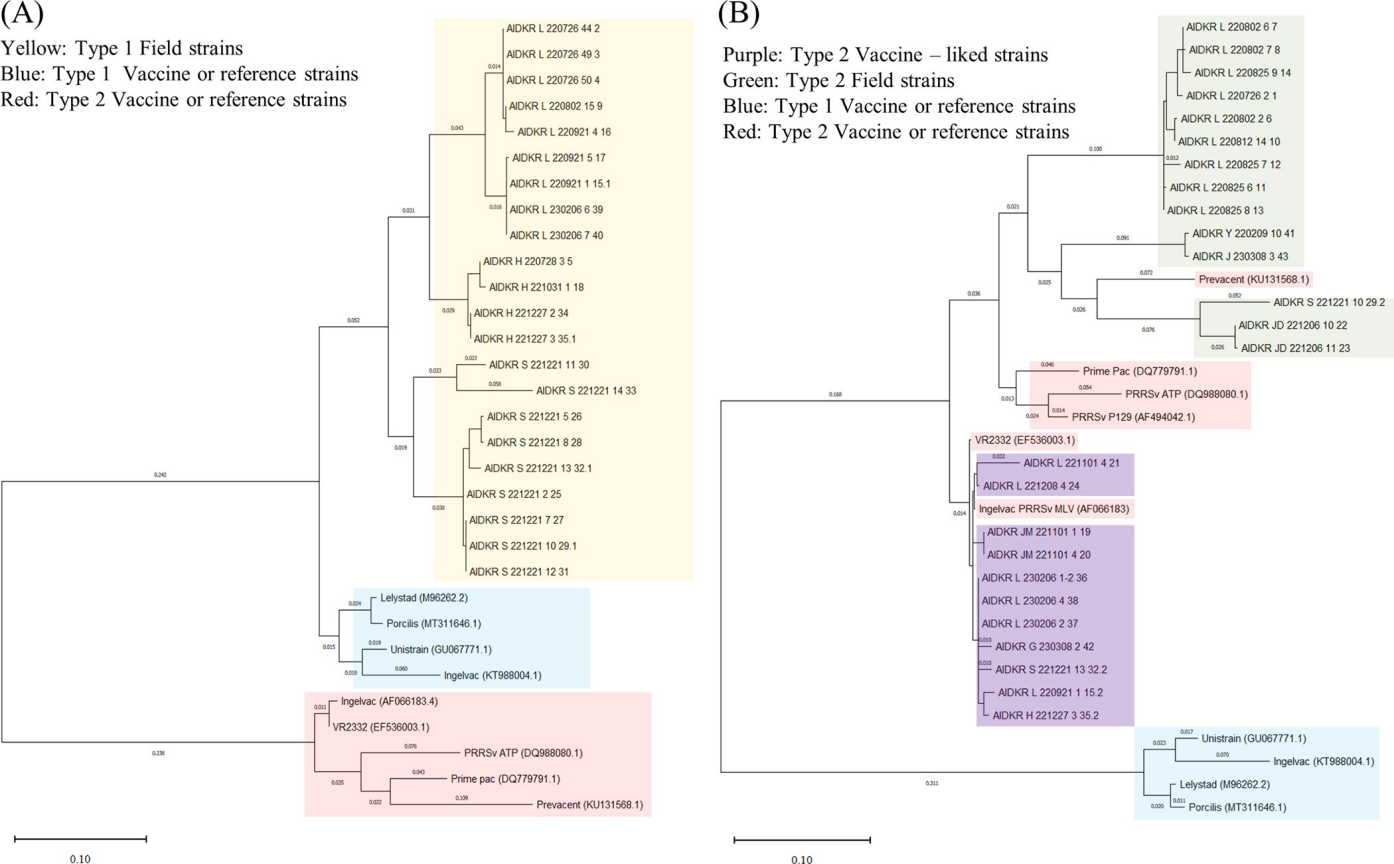
The phylogenetic tree analysis of the field samples of PRRSV-1 (A) and PRRSV-2 (B) using ORF5 Sanger sequencing results.

Evaluating the diagnostic agreement and performance of different assays is important in clinical diagnostics [42] Therefore, we assessed the diagnostic agreement and performance of the developed RT-qPCR assay with two commercial kits (Kit A and B) using 211 field samples. Forty-three field samples analyzed by ORF5 gene sequencing were used to compare the results of three RT-qPCR kits (Kit A, B, and the developed RT-qPCR assay; Fig 2 and Table 11). Sanger sequencing of the entire ORF5 gene of PRRSV in 43 samples from 8 farms out of 211 field samples identified 20 samples as PRRSV-1 and 23 as PRRSV-2, among which 4 were infected with both PRRSV-1 and -2 (Fig 2). Furthermore, all 20 PRRSV-1 samples were identified as PRRSV field strains (Fig 2A), wherein among the 43 PRRSV-2 samples, 10 were identified as PRRSV vaccine-like and the remaining 13 as PRRSV field strains (Fig 2B).

**Table 11:** Comparison of the results obtained from three RT-qPCR kits (Kit A, B, and the developed RT-qPCR assay) for field samples obtained using Sanger sequencing.

	Sample name*	Results of ORF5 Sanger sequencing	Kit A	Kit B	The developed RT-qPCR
1	AIDKR_L_220726_2_1	PRRSV-2	PRRSV-2	PRRSV-1/2	PRRSV-2
2	AIDKR_L_220726_44_2	PRRSV-1	PRRSV-1	PRRSV-1	PRRSV-1
3	AIDKR_L_220726_49_3	PRRSV-1	PRRSV-1	PRRSV-1	PRRSV-1
4	AIDKR_L_220726_50_4	PRRSV-1	PRRSV-1	PRRSV-1	PRRSV-1
5	AIDKR_H_220728_3_5	PRRSV-1	PRRSV-1	PRRSV-1	PRRSV-1
6	AIDKR_L_220802_2_6	PRRSV-2	PRRSV-2	PRRSV-2	PRRSV-2
7	AIDKR_L_220802_6_7	PRRSV-2	PRRSV-2	PRRSV-1/2	PRRSV-2
8	AIDKR_L_220802_7_8	PRRSV-2	PRRSV-2	PRRSV-1/2	PRRSV-2
9	AIDKR_L_220802_15_9	PRRSV-1	PRRSV-1	PRRSV-1	PRRSV-1
10	AIDKR_L_220812_14_10	PRRSV-2	PRRSV-2	PRRSV-2	PRRSV-2
11	AIDKR_L_220825_6_11	PRRSV-2	PRRSV-2	PRRSV-2	PRRSV-2
12	AIDKR_L_220825_7_12	PRRSV-2	PRRSV-2	PRRSV-1/2	PRRSV-2
13	AIDKR_L_220825_8_13	PRRSV-2	PRRSV-2	PRRSV-2	PRRSV-2
14	AIDKR_L_220825_9_14	PRRSV-2	PRRSV-2	PRRSV-2	PRRSV-2
15*	AIDKR_L_220921_1_15.1 AIDKR_L_220921_1_15.2	PRRSV-1/2	PRRSV-1/2	PRRSV-1	PRRSV-1/2
16	AIDKR_L_220921_4_16	PRRSV-1	PRRSV-1	PRRSV-1	PRRSV-1
17	AIDKR_L_220921_5_17	PRRSV-1	PRRSV-1	PRRSV-1	PRRSV-1
18	AIDKR_H_221031_1_18	PRRSV-1	Negative	PRRSV-1	PRRSV-1
19	AIDKR_JM_221101_1_19	PRRSV-2	PRRSV-2	PRRSV-1/2	PRRSV-2
20	AIDKR_JM_221101_4_20	PRRSV-2	PRRSV-2	PRRSV-1/2	PRRSV-2
21	AIDKR_L_221101_4_21	PRRSV-2	PRRSV-2	Negative	PRRSV-2
22	AIDKR_JD_221206_10_22	PRRSV-2	PRRSV-2	PRRSV-1/-2	PRRSV-1/2
23	AIDKR_JD_221206_11_23	PRRSV-2	PRRSV-2	PRRSV-1/2	PRRSV-2
24	AIDKR_L_221208_4_24	PRRSV-2	PRRSV-2	PRRSV-1/2	PRRSV-2
25	AIDKR_S_221221_2_25	PRRSV-1	Negative	PRRSV-1	PRRSV-1
26	AIDKR_S_221221_5_26	PRRSV-1	PRRSV-1	PRRSV-1	PRRSV-1
27	AIDKR_S_221221_7_27	PRRSV-1	PRRSV-1	PRRSV-1	PRRSV-1
28	AIDKR_S_221221_8_28	PRRSV-1	PRRSV-1	PRRSV-1	PRRSV-1
29*	AIDKR_S_221221_10_29.1 AIDKR_S_221221_10_29.2	PRRSV-1/2	PRRSV-1/2	PRRSV-1/2	PRRSV-1/2
30	AIDKR_S_221221_11_30	PRRSV-1	PRRSV-1	PRRSV-1	PRRSV-1
31	AIDKR_S_221221_12_31	PRRSV-1	PRRSV-1	PRRSV-1	PRRSV-1
32*	AIDKR_S_221221_13_32.1 AIDKR_S_221221_13_32.2	PRRSV-1/2	PRRSV-1/2	PRRSV-1/2	PRRSV-1/2
33	AIDKR_S_221221_14_33	PRRSV-1	PRRSV-1	PRRSV-1	PRRSV-1
34	AIDKR_H_221227_2_34	PRRSV-1	PRRSV-1	PRRSV-1/2	PRRSV-1
35*	AIDKR_H_221227_3_35.1 AIDKR_H_221227_3_35.2	PRRSV-1/2	PRRSV-1	PRRSV-1	PRRSV-1
36	AIDKR_L_230206_1–2_36	PRRSV-2	PRRSV-2	PRRSV-2	PRRSV-2
37	AIDKR_L_230206_2_37	PRRSV-2	PRRSV-2	PRRSV-1/2	PRRSV-2
38	AIDKR_L_230206_4_38	PRRSV-2	PRRSV-2	PRRSV-1/2	PRRSV-2
39	AIDKR_L_230206_6_39	PRRSV-1	PRRSV-1	PRRSV-1	PRRSV-1
40	AIDKR_L_230206_7_40	PRRSV-1	PRRSV-1/2	PRRSV-1	PRRSV-1
41	AIDKR_Y_220209_10_41	PRRSV-2	PRRSV-2	PRRSV-2	PRRSV-2
42	AIDKR_G_230308_2_42	PRRSV-2	Negative	PRRSV-1/-2	PRRSV-2
43	AIDKR_J_230308_3_43	PRRSV-2	PRRSV-2	PRRSV-2	PRRSV-2

*For samples in which both PRRSV-1 and -2 were detected, they were suffixed with .1 for PRRSV-1 and .2 for PRRSV-2.

A comparison of the sensitivity, specificity, and diagnostic agreement between the three assays based on Sanger sequencing results is shown in Table 12. The developed RT-qPCR showed a sensitivity of 96.0%, specificity of 100%, and diagnostic agreement of 97.7%. Our findings indicate that the developed RT-qPCR assay demonstrated higher diagnostic agreement, sensitivity, and specificity than both the commercial kits did (Tables 11 and 12).

**Table 12:** Comparison of sensitivity, specificity, and diagnostic agreement between the three assays based on Sanger sequencing results.

Diagnostic accuracy	PRRSV-1			PRRSV-2		
	Kit A	Kit B	The developed RT-qPCR	Kit A	Kit B	The developed RT-qPCR
Sensitivity	90.9%	100%	100%	91.0%	88.0%	96.0%
Specificity	100%	42.9%	95.2%	94.4%	94.4%	100%
Diagnostic agreement	95.3%	72.1%	97.7%	93.0%	90.7%	97.7%
PPV	100%	64.7%	95.7%	95.8%	95.7%	100%
NPV	91.3%	100%	100%	89.5%	85.0%	94.7%

Furthermore, we compared the results of Kit B and the developed RT-qPCR assays with those of Kit A (reference kit) for the 211 field samples (including the previous 43 field samples). As shown in Table 13, the developed RT-qPCR assay showed a higher sensitivity (100%), specificity (97%), diagnostic agreement (97.6%), PPV (89.4%), and NPV (100%) than Kit B (92.9%, 45.0%, 54.5%, 29.5%, and 96.2%, respectively) for PRRSV-1. Similarly, the sensitivity, diagnostic agreement, PPV, and NPV of the RT-qPCR assay for PRRSV-2 were higher than those of Kit B; however, the specificity of both kits was similar (Table 13).

**Table 13:** Comparison of sensitivity, specificity, and diagnostic agreement with Kit A.

Diagnostic accuracy	PRRSV-1		PRRSV-2	
	Kit B	The developed RT-qPCR assay	Kit B	The developed RT-qPCR assay
Sensitivity	92.9%	100%	75.0%	90.9%
Specificity	45.0%	97.0%	91.9%	91.9%
Diagnostic agreement	54.5%	97.6%	84.8%	91.5%
PPV	29.5%	89.4%	86.8%	88.9%
NPV	96.2%	100%	83.7%	93.4%

## Discussion

PRRS is a major endemic disease in pigs that has been occurring steadily not only in South Korea but also worldwide since it was first reported in 1987 [10, 43, 44]. In recent years, highly pathogenic PRRSV has become prevalent, causing considerable damage to the pig industry since 2013. Therefore, various measures, such as vaccination, biosecurity, diagnosis, and herd management strategies, have been implemented to prevent and control the spread of PRRSV. However, this remains a major problem for many pig producers [12, 45, 46].

Since PRRSV was discovered 30 years ago, various diagnostic methods have been developed to detect and identify this virus. These methods include viral culture for isolating and identifying PRRSV in samples suspected of harboring the virus, ELISA for detecting PRRSV-specific antibodies, and PCR-based methods for directly identifying PRRSV. However, each method has its own strengths and limitations, as discussed below [29, 47–52].

Virological diagnosis of PRRSV is particularly challenging, often necessitating isolation through a cell culture system. The advantage of isolation by culture is that it produces large amounts of virus, which increases diagnostic accuracy and has been regarded as the golden standard method [53]. Therefore, it is often preceded by other molecular diagnostic tests. Porcine alveolar macrophages and MARC-145 are the most susceptible cells for PRRSV [54–57]. However, the susceptibility can vary between batches of cells and viruses. For instance, field-isolated viruses, in particular, are generally present in low concentrations, and the susceptibility of the cell could be variable, leading to increased rates of culture failure [54, 57]. Furthermore, confirmation of PRRSV growth often requires subsequent RT-PCR analysis, which introduces the risk of contamination. This culture method is also time-consuming due to the extended incubation period, typically lasting one to two weeks [53].

ELISA is one of the most suitable tests for detecting PRRSV antibodies in numerous samples due to its cost-effectiveness, rapidness, and ease of performing [58]. However, ELISA has limitations in differentiating between positive results due to the **PRRS**V vaccine strain and **PRRS**V infection. Additionally, it cannot distinguish between infections caused by **PRRS**V**-1 and -2**, complicating the use of PRRSV vaccines for infection control [59–61]. Furthermore, ELISA is unsuitable for detecting early-stage infections, especially within 7 days post-infection, because PRRSV-specific antibodies are usually produced after 7 days post-infection [24, 58, 62, 63]. Among the PCR-based methods, RT-PCR amplifies specific regions of the PRRSV genome and subsequently detects the presence of amplicons and their sizes through gel electrophoresis. However, it tends to be less sensitive than other PCR methods because it requires a PCR product of 5 ng or more, which is the sensitivity of the gel. In addition, Sanger sequencing requires amplifying sites used to check the genetic diversity of PRRSV, such as ORF5, through RT-PCR, followed by checking them through a sequencer. While sequencing offers the advantage of precise analysis of PRRSV presence and genotype, it is often less sensitive than PCR due to the need for amplifying longer sequences. It also requires an RT-PCR and sequencing, making it more time-consuming and expensive.

Among all diagnostic methods, RT-qPCR is the easiest and fastest tool to diagnose PRRSV infection. The advantages of RT-qPCR include the following: RT-qPCR has gained widespread acceptance for PRRSV detection due to its speed, high sensitivity, reproducibility, and reduced risk of contamination. This method utilizes a specific probe to capture the short amplicons generated by RT-PCR, resulting in considerably shorter PCR time and high sensitivity. In addition, RT-qPCR differentiates between PRRSV-1 and -2 with remarkable sensitivity and specificity [24]. However, RT-qPCR also has several limitations. First, it relies on the presence of the PRRSV antigen in the blood sample, and there are reports that the probability of diagnosis drops below 50% more than six weeks after infection, indicating that PRRSV may not be detectable approximately six weeks after infection [24]. To overcome this problem, it is recommended that pigs be tested for PRRSV infection upon introduction into the farm or regularly. However, determining the exact timing of PRRSV infection is difficult, which can affect the diagnostic accuracy. In addition, RT-qPCR encounters challenges owing to the genetic diversity of PRRSV. The high mutation rate of PRRSV leads to the accumulation of genetic variation over time, which makes it challenging to diagnose using conventional RT-qPCR. This is caused by the increased genetic variation in PRRSV, which can lead to potential mismatches between the primers/probes and target sequences. Consequently, there is an increased risk of false-negative results due to increased undiagnosable PRRSV strains. Therefore, it is necessary to continuously monitor the changes in PRRSV genotypes and develop suitable RT-qPCR methods for accurate detection. Despite the drawbacks of RT-qPCR, researchers have dedicated efforts to advance and enhance its utility as a method for diagnosing PRRSV because of its superior reliability compared with that of other diagnostic approaches [24, 64].

To diagnose PRRSV using RT-qPCR, we thoroughly analyzed a substantial number of PRRSV sequences available in the NCBI database and successfully identified highly conserved regions specific to PRRSV. These regions were used to develop a novel diagnostic method for PRRSV, which we presented in this study. The results obtained using the developed RT-qPCR assay demonstrated strong agreement with those obtained using Kit A and ORF5 Sanger sequencing. However, the concordance between the developed RT-qPCR assay and Kit B is relatively low. Nevertheless, considering the high concordance observed in the results of Kit A and the developed RT-qPCR with ORF5 sequencing, it can be inferred that Kit B is more likely to produce false-positive or false-negative results than the other two methods.

Our study has three limitations. First, for field samples in which ORF5 sequencing was not feasible, additional confirmation could not be conducted when there were discrepancies between the results obtained from the two commercial kits and the developed RT-qPCR assay. Consequently, these conflicting results cannot be verified. Therefore, the actual sensitivity of each method may have been either underestimated or overestimated because of these limitations. However, because Kits A and B are currently certified by KAPQA, it is less likely that there were notable errors in the comparative results themselves, and it is more likely that the differences were due to genetic variations in PRRSV. Second, RNA extraction from the 211 PRRSV samples obtained in the field was performed directly without prior culturing, which may have influenced the sensitivity, specificity, and diagnostic concordance assessment. For instance, when comparing the results of the developed RT-qPCR assay with ORF5 sequencing, there was one false-negative and one false-positive result. The false-negative result was slightly higher than the Ct value of 40, which was considered negative. This result may be attributed to the low RNA concentration in the sample because of direct RNA extraction without culturing, which could have increased the probability of human error during the evaluation process. Finally, the number of field samples used to compare the performance of the developed RT-qPCR was small. Due to the small number of samples, the statistical error and bias may have been greater and not sufficient for accurate comparative evaluation. Especially in the case of PRRSV, which has a high mutation rate, it is necessary to conduct comparative evaluations with a larger number of field samples and field samples collected from various regions to fully reflect the genetic diversity of PRRSV.

Despite the present study’s limitations, the implications of our developed assay hold significant promise for PRRSV diagnosis and, therefore, its control and prevention in the swine industry. By enhancing the sensitivity of the diagnostic method, we can enable early detection of PRRSV infections, even in cases with low viral loads. This early detection empowers veterinarians and producers to swiftly implement targeted intervention strategies, reducing the potential for widespread transmission and minimizing the impact of PRRSV infection. As a result, the developed assay can play a pivotal role in reducing disease-related losses and safeguarding the health of swine populations. Moreover, the improved specificity of the assay ensures accurate differentiation between PRRSV-1 and PRRSV-2 subtypes. This specificity is essential for implementing precise control measures. By accurately identifying the PRRSV subtype, we can optimize treatment protocols and reduce the risk of misdiagnosis, further enhancing disease management strategies.

In addition, in the future, we will evaluate the developed RT-qPCR through continuous field sample collection. By testing more field samples verifying the sensitivity, specificity, and inter-experimenter reproducibility of the developed RT-qPCR, a suitable PRRSV diagnostic will be possible. Further research will be conducted to study the genetic diversity of PRRSV outbreaks in South Korea through whole genome sequencing to understand the genetic evolution of PRRSV and apply it to optimize the PRRSV diagnostic method further.

In summary, we successfully developed an RT-qPCR assay for PRRSV detection that demonstrated comparable or better performance than conventional assays, as evaluated by comparison with ORF5 sequencing and commercial kits certified by the KAPQA. The developed assay is anticipated to contribute to PRRSV diagnosis, thereby facilitating the effective control of PRRSV in the swine industry.

## Supporting information

S1 Fig Representative agarose gel electrophoresis result of PCR products using two primer sets for PRRSV-1 and -2 ORF5 sequencing. M is the DNA ladder; lane 1, Unistrain^®^ (HIPRA, Girona, Spain) of PRRSV-1; Fostera^®^ (Zoetis Inc., NJ, USA) of PRRSV-2; Lanes 2 and 4; DW (Negative). Samples in lanes 1 and 2 were amplified using a primer set specific to PRRSV-1, and those in lanes 3 and 4 were amplified using PRRSV-2-specific primers.
(PDF)

S2 Fig The whole genome map of PRRSV and primer and probe locations of the developed RT-qPCR. The genome map of PPRSV was constructed using SnapGene (ver.7.0.2) and further modified to represent the average length of the PRRSV strains. Primer and probe information is shown using the sequences from Lelystad (M96262.2) for PRRSV-1 and VR2332 (EF536003.1) for PRRSV-2.
(PDF)
