# Supplementary figures and images for: Development of a one-step reverse transcription-quantitative polymerase chain reaction assay for the detection of porcine reproductive and respiratory syndrome virus
Source: PLoS One. 2023 Oct 16;18(10):e0293042. doi: 10.1371/journal.pone.0293042 (PMC10578580; doi:10.1371/journal.pone.0293042)

**S1 Fig. Gel electrophoresis result of PCR products for ORF5 sequencing.**

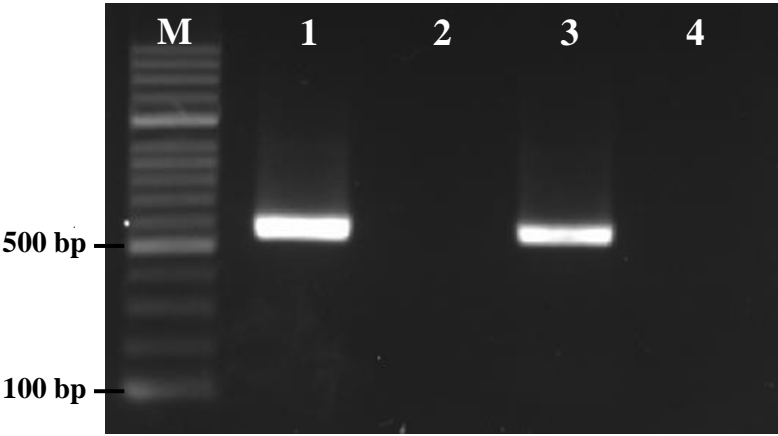

Supplement: S1 Fig — M is the DNA ladder; lane 1, Unistrain® (HIPRA, Girona, Spain) of PRRSV-1; Fostera® (Zoetis Inc., NJ, USA) of PRRSV-2; Lanes 2 and 4; DW (Negative). Samples in lanes 1 and 2 were amplified using a primer set specific to PRRSV-1, and those in lanes 3 and 4 were amplified using PRRSV-2-specific primers. (PDF) [file pone.0293042.s001.pdf]

**S2 Fig. The whole genome map of PRRSV and primer and probe locations of the developed RT-qPCR**

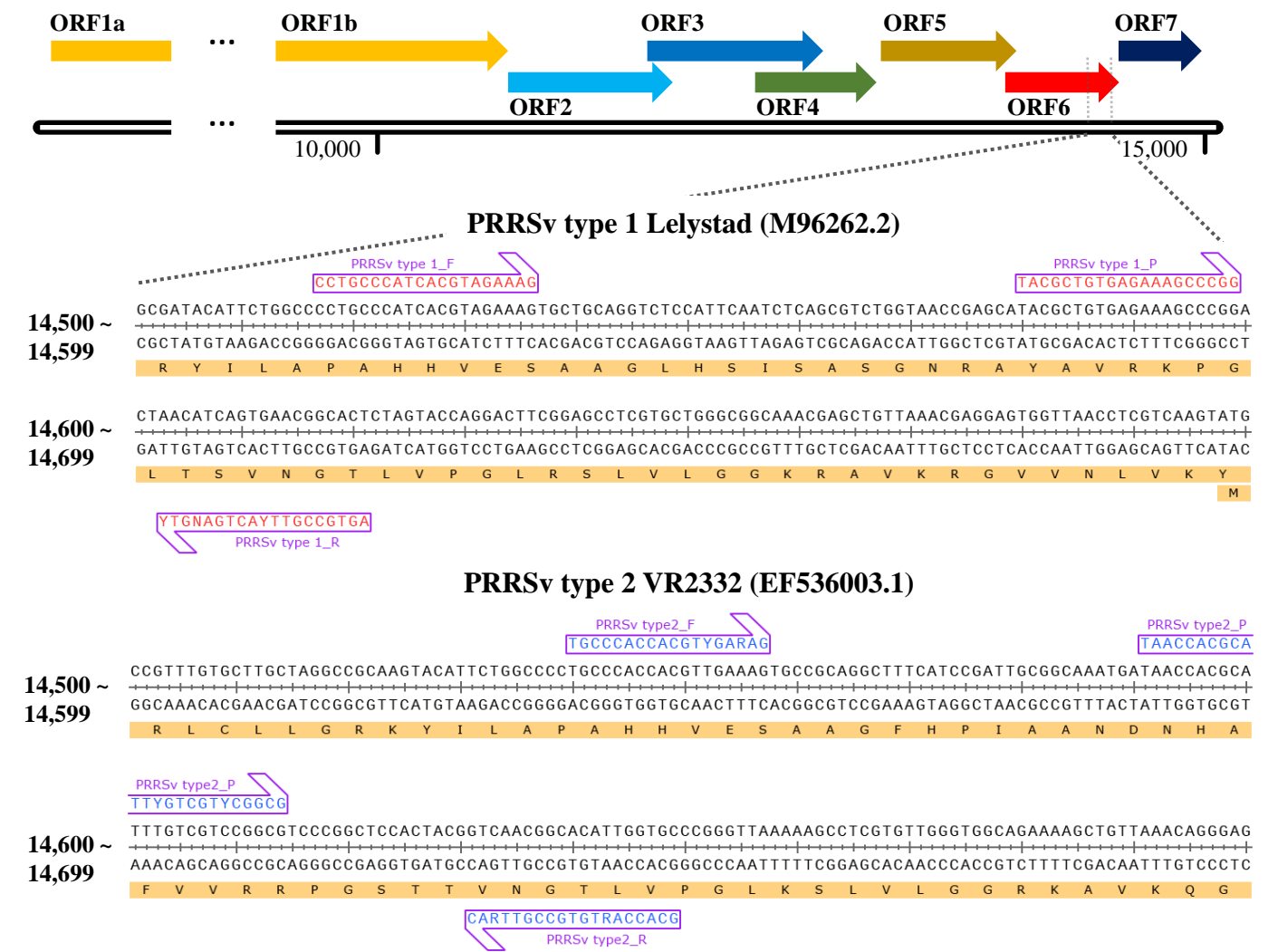

Supplement: S2 Fig — The genome map of PPRSV was constructed using SnapGene (ver.7.0.2) and further modified to represent the average length of the PRRSV strains. Primer and probe information is shown using the sequences from Lelystad (M96262.2) for PRRSV-1 and VR2332 (EF536003.1) for PRRSV-2. (PDF) [file pone.0293042.s002.pdf]
